# Communication when bone and joint infection in children is suspected: a qualitative study of patients, families, and health professionals

**DOI:** 10.1302/2633-1462.72.BJO-2025-0179.R1

**Published:** 2026-02-20

**Authors:** Andrew Kirkcaldy, C. V. E. Carpenter, Nicolas Magrane, Marie-Caroline Nogaro, Anjali Shah, Daniel C. Perry, Tim Theologis, Bridget Young

**Affiliations:** 1 Department of Public Health, Policy and Systems, University of Liverpool, Liverpool, UK; 2 Bristol Medical School, University of Bristol, Bristol, UK; 3 Medicines and Healthcare Products Regulatory Agency, London, UK; 4 Nuffield Department of Orthopaedics Rheumatology and Musculoskeletal Sciences, University of Oxford, Oxford, UK; 5 Institute of Life Course & Medical Sciences, University of Liverpool, Liverpool, UK

**Keywords:** Paediatrics, Bone and joint infection, Diagnosis, Communication, Qualitative research, Interviews, joint infection, MRI scan, diagnostic testing, anxieties, radiographs, joint damage, blood, sepsis, paediatric orthopaedic surgeons, infection

## Abstract

**Aims:**

Bone and joint infection in children can result in bone and joint damage, sepsis, and even death. Diagnosis is informed by the history, examination, and a suite of tests, including blood markers, radiographs, ultrasound, and MRI. This study aimed to identify the communication needs of families during diagnostic assessment of bone and joint infection.

**Methods:**

This was a qualitative study involving semi-structured interviews with children and families who had experienced diagnostic tests for bone and joint infection, and health professionals experienced in the care of affected children. A total of 21 families (four children; 21 parents) and 11 health professionals from 11 English and Welsh hospitals were interviewed. Data analysis was informed by thematic analysis.

**Results:**

Families often felt highly anxious during diagnosis. Some described a disorderly diagnosis process, gaps or inconsistencies in information, and insensitive communication that contributed to their anxiety. Other families described more positive experiences, indicating how health professionals helped them feel prepared by providing an outline of timelines, the rationale for tests, and the potential need to adjust plans as new information became available. Participants recognized the importance of age-appropriate communication with children, and the involvement of play specialists in this process.

**Conclusion:**

The findings demonstrate the intense anxiety families experience during assessment of bone and joint infection, particularly when they are left to make sense of uncertainties by themselves, or when communication is unclear. Health professionals can support families during diagnosis by attending to both the information they provide and how it is provided, while acknowledging the uncertainties of the diagnosis process. Specifically, families valued explanations of the rationale for different clinical investigations and their timing, and advance discussion of the next steps and possible outcomes of diagnostic testing. When test results became available, families were also helped by clear follow-up plans, including in situations when the results were inconclusive.

Cite this article: *Bone Jt Open* 2026;7(2):259–265.

## Introduction

Each year approximately 1,800 children in England are admitted to hospital with bone and joint infection (BJI).^1^ This is caused by a bacterial infection of the bone that is typically introduced or spread through the bloodstream.^2,3^ BJI often presents with symptoms of local pain, difficulty bearing weight, fatigue, high temperature, and erythema,^4,5^ and infection can spread into the adjacent joint and cause septic arthritis. Osteomyelitis and septic arthritis are intricately linked in children, and are usually considered together as BJI. These can lead to irreversible joint damage, bone destruction, and systemic sepsis.^3,6^ Prompt diagnosis, through clinical assessment, blood tests, and imaging, is paramount,^6^ although UK-based studies have noted challenges with the availability and access to some diagnostic tools, including limited out-of-hours MRI services^7^ and inconsistencies in access to ultrasound.^8^

Studies of BJI in children have typically focused on the route to diagnosis and treatment, and while there is evidence that parents prioritize receiving a quick and accurate diagnosis,^9^ little attention has been given to the wider experiences of patients and families and their needs during diagnosis. We report on a qualitative study investigating patient, family, and health professional experiences of communication during the diagnostic process for BJI, from the point of help-seeking. Qualitative methods generate detailed, contextual data and insights to inform care in health settings.^10,11^ They are particularly well suited to explore participants’ experiences^12^ and to identify ways to improve these.^13^

## Methods

### Design

This qualitative interview study was nested within the broader Imaging in Paediatric Osteomyelitis (PICBONE) study.^3^

### Sample and recruitment

Interview participants were children (aged 7 to 15 years) who had undergone diagnostic tests for BJI, parents of children with experience of these tests, and health professionals involved in the care of children with suspected BJI. Participants were recruited from 11 English NHS hospital trusts and a Welsh health board. English NHS trusts were based in six of the country’s seven NHS regions. Parents were initially approached about the study when their child presented to hospital. A total of 290 parents agreed to be contacted about the study. A sample of these parents were contacted by the qualitative researcher, AK, between October 2023 and July 2024. While we predominately sampled families with direct experience of a BJI diagnosis, we also sampled a subset who had been assessed but not diagnosed with BJI, as our focus was on the experience of communication from presentation with suspected BJI. Using Indices of Multiple Deprivation (IMD) deciles as an indicator of relative deprivation, we strove for a socioeconomically diverse sample.^14^ After scheduling each interview, parents were asked to consent to their own and, if applicable, their child’s participation. The assent of children was confirmed prior to each interview.

Health professionals were identified through representatives of the PICBONE steering group, study management group, associate principal investigators (PIs), and designated study PIs within each NHS organization. Those identified were contacted by the qualitative researcher, and their informed consent was recorded before each interview.

### Participant characteristics

Of the families contacted (n = 86), 21 (21 parents and four children) were interviewed. Among participating families, 13 children were aged under five years when investigated for BJI, while eight were aged five to 15 years. Overall, 19 parents and two children were interviewed individually, while two parents and two children were interviewed together; individual parental interviews averaged around 30 minutes, and interviews with children averaged around ten minutes. A total of 15 families were interviewed within three months of their first consultation with a health professional, while the remainder were interviewed within six months. Of the 21 families, 16 had experience of a confirmed diagnosis of BJI and five had experience of BJI investigation without a subsequent diagnosis of infection. IMD deciles indicated that three families lived in neighbourhoods of relatively high deprivation (deciles 1 to 3), ten lived in mid-range neighbourhoods (deciles 4 to 7), and seven lived in neighbourhoods of relatively low deprivation (deciles 8 to 10), with data unavailable for one family. In terms of ethnicity, our sample included two participants who identified as Black, two as Mixed ethnicity, and 21 as White. Key characteristics of parents and children are presented in Table I.

**Table I.** Key characteristics of family participants.

Parents					
Participant code	Relationship to child admitted to hospital	Age of child, yrs	Ethnicity	IMD decile*	BJI infection diagnosed
F1P	Mother	6	White	7	N
F2P	Mother	1	White	3	Y
F3P	Mother	3	White	8	N
F4P	Mother	6	White	5	N
F5P	Mother	1	Mixed	9	Y
F6P	Mother	1	White	8	Y
F7P	Mother	1	White	7	N
F8P	Mother	1	Mixed	7	Y
F9P	Father	5	White	10	N
F10P	Mother	1	White	2	Y
F11P	Mother	8	White	4	Y
F12P	Father	1	White	4	Y
F13P	Mother	15	White	10	Y
F14P	Father	7	Black	2	Y
F15P	Mother	< 1	White	Unknown	Y
F16P	Mother	1	White	9	Y
F17P	Mother	14	White	4	Y
F18P	Mother	1	White	7	Y
F19P	Mother	< 1	White	4	Y
F20P	Mother	3	White	9	Y
F21P	Father	15	White	4	Y
**Children**					
**Participant** **code**	**Sex**	**Age, yrs**	**Ethnicity**	IMD decile*	**BJI infection**
F13C	Male	15	White	10	Y
F14C	Male	7	Black	2	Y
F17C	Female	14	White	4	Y
F21C	Male	15	White	4	Y

* Lower decile indicates family living in area of higher relative deprivation.

BJI, bone and joint infection; IMD, Indicies of Multiple Deprivation.

### Data collection

Semi-structured interviews took place via Microsoft Teams (Microsoft, USA) (n = 6) or telephone (n = 28) between November 2023 and July 2024, and were digitally audio-recorded. AK, a postdoctoral researcher with previous training and experience of qualitative research, conducted all interviews. Before each interview, AK informed participants that his background was non-clinical, that he was independent of the clinical teams at each hospital, and reassured participants of their anonymity. Interview topic guides (Supplementary Material) were developed by the research team, with input from the study management team and steering group, including patient and public involvement (PPI) contributors. Interviews with parents and children explored their views and experiences of the diagnostic pathway for BJI, including experiences of different tests (blood test, radiograph, MRI, ultrasound) and receiving the results of these tests. Health professionals were interviewed about their experiences of providing health care to those with suspected BJI (Supplementary Material). Interviews ceased when data saturation had been reached across the parents’ and health professionals’ interviews, and no new themes were being identified from these data. Due to the low numbers of children with suspected infection who met the minimum age for interview eligibility, we were unable to interview as many children as expected. Thus, we do not believe saturation was reached for their data.

### Data analysis

Pseudonymized transcripts of interviews were imported into NVivo 14 for Windows (Lumivero, USA). Interpretative data analysis was led by AK and informed by Braun and Clark’s^15^ six-step framework for thematic analysis: 1) data familiarization through multiple readings of each transcript; 2) coding the dataset; 3) searching for themes; 4) reviewing themes; 5) defining themes; and 6) writing. Each stage involved regular meetings between the study’s qualitative researchers AK and BY, to review the developing analysis and consider different interpretations of the data. Both AK and BY were experienced in qualitative health research, although neither had a background as a clinical professional. Therefore, CVEC, a clinical lecturer with a background in orthopaedics and experience of qualitative research, also contributed to the analysis to help ensure that data interpretation was clinically informed.

## Results

Health professionals interviewed (n = 11) included a paediatric radiologist, a registrar in paediatric orthopaedic surgery, a play specialist, a paediatric emergency consultant, paediatric nurses (n = 2), radiographers (n = 3), and consultant paediatric orthopaedic surgeons (n = 2) (Table II). Their average interview length was 30 minutes. Qualitative findings for both families and health professionals are presented in two overarching themes: 1) clarity and consistency of communication, and 2) manner of communication. Quotes from participants’ interviews are presented to illustrate the findings, with brief quotes usually integrated in the main text, while longer quotes are shown in Table III.

**Table II.** Key characteristics of health professionals.

Participant code	Job title	Sex	Ethnicity
HP1	Paediatric Radiologist	Female	White
HP2	Registrar (Paediatric Orthopaedics)	Female	White
HP3	Consultant Paediatric Orthopaedic Surgeon	Male	White
HP4	Consultant Paediatric Orthopaedic Surgeon	Male	White
HP5	Paediatric Nurse	Female	White
HP6	Paediatric Nurse	Male	White
HP7	Play Specialist	Female	White
HP8	Paediatric Emergency Consultant	Female	Mixed
HP9	Radiographer	Female	White
HP10	Radiographer	Female	White
HP11	Radiographer	Female	White

**Table III.** Illustrative quotes from participant interviews.

Quote code	Illustrative quote
Q1	“She spent two weeks in hospital… I wasn’t told until… the backend of the first week what it [the diagnosis] might be” (F9P)
Q2	*“*Maybe just more ideas of times when things were going to happen” (F2P)
Q3	”It just felt like we were being sent for more tests without any explanation as to why… we felt completely in the dark” (F6P)
Q4	“We were wondering why she wasn't having an ultrasound and why they were doing an X-ray… our minds were just running away with us at that point” (F6P)
Q5	“They were quite on the ball of testing, checking and updating [us] constantly… this one may not work so they'd have to check something else. So… it wasn't surprise news” (F14P)
Q6	“They give you the impression that you're going to be seen quicker… and then obviously you're not” (F16P)
Q7	“They had told me to stop breastfeeding two hours before I actually really had to… which obviously made quite a difference for [my daughter] and for me” (F10P)
Q8	*“*She was giving us the relevant information we needed to know at that time… she would sort of drip feed a kind of a possibility, but it wasn't done in a way that… [was] scary. It was very much… just so you know, this is the possibility for the next step… I wasn't worrying because she'd already primed us” (F20P)
Q9	“This could be a joint infection, it can turn out quite nasty we really need to get on top of it” (HP5)
Q10	“Teenagers, will understand a lot more than a five-year-old or two-year-old would” (HP9)
Q11	“I will ask [the child] a question and I will try to give them the time to put into words what they want to ask… [it’s about] getting him to tell me what he feels himself rather than the mum or dad putting their views onto the child” (HP7)
Q12	“[He spoke] in a really child-friendly way. He didn't use big scary words. He kept [him] informed, but for a six-year-old” (F4P)
Q13	“[Play specialists]… know how to talk to them [children], they know how to make them feel at ease” (HP2)
Q14	“[The play specialist]… was really sweet. She takes a 3D model and explains how it works… it made me more comfortable knowing what might happen” (F17C)
Q15	“I mentioned that his legs were cold to the touch, they said… ‘that’s nothing’, it was kind of dismissive*”* (F12P)
Q16	“My wife said that she wasn’t going to leave until they took bloods” (F12P)
Q17	“I'm wanting… updates… instead I'm actually just having to relay history… doesn't fill you with confidence” (F10P)
Q18	*“*I feel like the notes should have been read… it’s like they weren't communicating” (FP5)
Q19	*“*This is the only criticism I have… you have to take on what the parent has said. They did after a while, but [initially] they would always come in… trying to comfort [my son] and I'm saying ‘Don’t, don't speak softly to him, he needs to hear this needs to be done, let’s get it done’” (F14P)

### Clarity and consistency of communication

All parents reported that their children underwent one or more tests (blood test, radiograph, MRI, ultrasound). While their descriptions of the diagnostic pathway differed, families often remarked on lengthy waits for tests, repeat visits, and hospital admissions and hospital transfers. Many parents recounted having felt intensely “anxious” or “worried” about their child’s condition during this time, “she was so [sic] in so much pain and no one would do anything” (F11P), with some expressing concern that their child’s symptoms might indicate “something sinister” (F18P) or “be an early sign of cancer” (F3P). In this context, families recurrently commented upon the importance of communication with health professionals, particularly the need for communication that was clear and consistent and helped families feel prepared for the next steps.

Families were often unaware of how long tests and diagnosis could take (Q1) and wanted health professionals to provide a clear picture of the anticipated diagnostic journey and the projected timeline (Q2). Parents also spoke about the importance of health professionals explaining the rationale for diagnostic tests. For example, a mother described her and her husband’s frustration at not having been told why her daughter needed an X-ray (Q3). She elaborated on the worry and distress that arose from not knowing why certain tests were being carried out (Q4). Reflecting on a subsequent hospital visit, the same mother described a very different experience of communication, remarking on having felt “reassured… because we had a plan, we knew which investigations we were having done and why*”* (F6P). This was echoed by another parent who spoke positively about “being informed” (F14P) about his child’s tests and the possibility that plans may need to change (Q5).

Families often highlighted inconsistencies in the information they received from healthcare professionals. Those who first presented at community services (the NHS 111 non-emergency medical helpline; GP practice), rather than hospital emergency departments, typically reported that communication between these services and hospitals, seemingly intended to speed up diagnosis, was ineffective (Q6). This same parent also described receiving contradictory instructions before her son’s MRI scan, with hospital staff initially informing that her son “wasn’t allowed to have his first nap until he had the MRI*”,* but later said “he needs to be asleep so that we can take him for the MRI*”* (F16P). A different mother, also recalling instructions provided prior to an MRI scan, recounted being told to stop breastfeeding earlier than necessary, which made comforting her infant unnecessarily difficult (Q7). Parents also described confusing communications about hospital appointments. While on her way to one hospital for an ultrasound scan, a mother received a telephone call from another hospital advising her not to attend for the appointment because of staff illness. Perplexed, she telephoned the original hospital to clarify: “So, then I was ringing [Hospital two] to say ‘Have I got an appointment?’ and they said ‘Yes… it’s still on’” (F1P). Information often appeared inaccurate and inconsistent to parents, which left them feeling the diagnostic journey was disorderly and exacerbated their sense of uncertainty. However, in instances where communication was accurate, informative, consistent, and timely, parents felt reassured and prepared for what was likely to happen and why (Q8).

Health professionals also commented on the importance of clarity when communicating with families, including the need to “ensure that the family and child are on board with the reasons [we] are doing the investigations” (HP4) and to be “upfront” with parents (Q9). Nevertheless, several alluded to the challenges of communication in the context of uncertainty, when the diagnosis is “not 100% conclusive on investigations or clinically” (HP8). One health professional spoke of how this uncertainty meant there was a need to balance being *“*open about my thoughts*”* with parents while avoiding creating *“*unnecessary anxieties until we've got a secure diagnosis” (HP3).

### Manner of communication

Though age influenced how and to what extent health professionals communicated with children (Q10), health professionals emphasized the importance of having direct one-to-one interactions with children where possible (Q11). Such an approach was clearly supported by families. A 14-year-old patient spoke favourably about hospital staff who “mainly” talked to her, rather than her parents, “it made me feel more like an adult… it helped out quite a bit” (F17C)*,* and a mother appreciatively recounted the way in which a consultant spoke to her son (Q12). When discussing the ways in which children were prepared for different diagnostic tests and subsequent care, health professionals emphasized that play specialists understood children and how to calm them (Q13). *S*upporting this assertion, an older child reflected on how a play specialist had helped her to feel “more comfortable” (F17C) about an MRI scan she was due to have (Q14).

Several families were less positive about the way communication with health professionals played out. A few spoke of being frightened by health professionals’ choice of words, such as a parent who recalled a doctor abruptly informing the family that they would likely have to stay in hospital for a long time: “he kind of scared me*”* (F5P). Another was alarmed by a doctor’s use of the term “blue lighted*”* when relaying that her infant was to be transferred by ambulance to another hospital: “you hear blue light and you think it’s a life-or-death emergency situation” (F11P). In contrast, a parent was angered when a doctor suggested the family should not have attended hospital as their child was not ill enough: “I thought that was extremely rude… I was angry.” (F8P). Others felt their concerns were not taken seriously (Q15), while several families described having to be insistent in their dealings with staff (Q16) or regretted that they had not been more assertive. After long waits, several parents noted their exasperation over having to prompt staff to provide pain relief for their child or repeatedly having “to go and ask*”* (F7P) for updates. Having to recount the same information to different health professionals was also a source of concern (Q17), with parents commenting that health professionals should have already been up to date (Q18). Others reported being unsure about their main point of contact during their child’s hospital stay and felt that having one person responsible for communicating with them “might have made things a bit easier*”* (F15P). A final suggestion, made by a father reflecting on blood being taken from his seven-year-old son, underlined the importance of health professionals acknowledging parents’ views on how best to communicate with their children (Q19).

## Discussion

This is the first study to focus on parent and child experiences of communication in cases of suspected BJI, and to incorporate the views of health professionals. The findings highlight the importance of clear and consistent family-centred communication during the diagnostic pathway, and complement work in other health contexts in emphasizing the importance of communication in paediatric healthcare and its influence on families’ satisfaction and experience.^16-18^ Our findings are important in drawing attention to families’ experience of BJI diagnosis as an often disjointed and disorderly process, while echoing previous work,^9,19,20^ in pointing to the anxiety, distress, and frustration that patients and families can experience during diagnostic journeys.

The diagnosis of conditions such as BJI is a complex process with many moving parts, complexities, and inherent uncertainties that make communication challenging for health professionals.^21^ Patients and families often perceive this uncertainty as a threat.^22^ There are various recommendations to assist health professionals in communicating in uncertain contexts,^23^ several of which address communicating uncertainty during diagnosis, and these broadly align with the perspectives of the families interviewed. For example, recommendations point to the importance of health professionals discussing potential scenarios and possible outcomes of diagnostic testing with patients, and providing insight into health professionals’ reasoning. The recommendations also emphasize the need to avoid leaving patients to make sense of uncertainty by themselves, and the importance of offering clear follow-up plans, particularly when a test result is uncertain.^23,24^

The current study points to the challenges for parents in managing children’s needs during diagnosis. This is particularly related to pain management, but also to feeding and sleep scheduling for younger children, along with how these challenges can be exacerbated by gaps or inconsistencies in information provision. Our findings complement work which draws attention to the value of both direct and developmentally appropriate communication between health professionals and children, and the involvement of play specialists to communicate with children.^25,26^

A strength of this study was the inclusion of families from multiple hospitals who had experience of a range of tests, including families with a positive diagnosis of BJI and those for whom BJI was excluded. Interviews with a range of health professionals provided an understanding of communication issues from their perspectives. The inclusion of participants from across England and Wales suggests that the findings are broadly transferable. Nevertheless, our study has limitations. We would have liked to interview more children, but most patients presenting with suspected infection were below the minimum age for interview eligibility, so our insights into their first-hand experiences are limited. Similarly, a relatively small number of participants were from minority ethnic groups or socioeconomically disadvantaged backgrounds, so future studies might address these shortfalls. In addition, future studies should involve a wider representation of health professionals involved in the investigation and management of BJI, such as paediatricians and paediatric infectious disease specialists.

In conclusion, families often experience diagnostic tests for suspected BJI as stressful, uncertain, and disorderly. This uncertainty and disorderliness can be alleviated by health professionals being clear and consistent in their communication, providing information to prepare families for the diagnostic journey and ensuring that families are not left to make sense of the inherent uncertainties of the process by themselves. Preparatory information that was helpful for families included explanations of the rationale for, and timing of, diagnostic tests and discussion of the possible outcomes of such tests. When test results became available, families were helped by clear communication about treatment or follow-up plans, including in situations when a result was inconclusive. Our findings indicate that families need to feel heard by health professionals, who consistently and calmly address their concerns throughout diagnosis and work to communicate in age-appropriate communication with children. For families facing the possibility of serious illness such as BJI, the findings show how fundamental the words and manner of health professionals are in shaping their experience.

**Take home message**

- Families often experience the bone and joint infection (BJI) diagnostic process as stressful, uncertain, and disjointed.

- Clinicians can help families through this process by proactively explaining the rationale and timing of diagnostic tests, discussing potential outcomes before testing, and sharing their clinical reasoning with families.

- This study makes it clear that good communication is not just about patient satisfaction—it is a clinical tool for reducing distress and helping families cope more effectively during the diagnostic process for suspected BJI.

## Data Availability

The data that support the findings for this study are available to other researchers from the corresponding author upon reasonable request.
